# The Role of TonB Gene in *Edwardsiella ictaluri* Virulence

**DOI:** 10.3389/fphys.2017.01066

**Published:** 2017-12-18

**Authors:** Hossam Abdelhamed, Mark L. Lawrence, Attila Karsi

**Affiliations:** Department of Basic Sciences, College of Veterinary Medicine, Mississippi State University, Mississippi State, MS, United States

**Keywords:** *tonB*, iron, virulence, ESC, channel catfish

## Abstract

*Edwardsiella ictaluri* is a Gram-negative facultative intracellular pathogen that causes enteric septicemia in catfish (ESC). Stress factors including poor water quality, poor diet, rough handling, overcrowding, and water temperature fluctuations increase fish susceptibility to ESC. The TonB energy transducing system (TonB-ExbB-ExbD) and TonB-dependent transporters of Gram-negative bacteria support active transport of scarce resources including iron, an essential micronutrient for bacterial virulence. Deletion of the *tonB* gene attenuates virulence in several pathogenic bacteria. In the current study, the role of TonB (NT01EI_RS07425) in iron acquisition and *E. ictaluri* virulence were investigated. To accomplish this, the *E. ictaluri tonB* gene was in-frame deleted. Growth kinetics, iron utilization, and virulence of the *Ei*Δ*tonB* mutant were determined. Loss of TonB caused a significant reduction in bacterial growth in iron-depleted medium (*p* > 0.05). The *Ei*Δ*tonB* mutant grew similarly to wild-type *E. ictaluri* when ferric iron was added to the iron-depleted medium. The *Ei*Δ*tonB* mutant was significantly attenuated in catfish compared with the parent strain (21.69 vs. 46.91% mortality). Catfish surviving infection with *Ei*Δ*tonB* had significant protection against ESC compared with naïve fish (100 vs. 40.47% survival). These findings indicate that TonB participates in pathogenesis of ESC and is an important *E. ictaluri* virulence factor.

## Introduction

Enteric septicemia of catfish (ESC) was first detected in the southern United States in 1976, and the disease was described in 1979 (Hawke, 1979). The etiologic agent of ESC is *Edwardsiella ictaluri*, which is in the family *Enterobacteriaceae* (Hawke et al., 1981). It is a facultative anaerobe that is motile with peritrichous flagella (Plumb and Sanchez, 1983). In its acute form, ESC causes gastroenteric septicemia, and its chronic form causes meningoencephalitis (Shotts et al., 1986; Newton et al., 1989) in cultured channel catfish (*Ictalurus punctatus*). Outbreaks of ESC occur during early summer and autumn, and fish are more at risk when water temperatures range from 22 to 28°C (Francis-Floyd et al., 1987). Stress and poor management practices increase susceptibility to ESC through alteration of host-defense mechanisms (Hawke and Khoo, 2004; Small and Bilodeau, 2005; Cunningham et al., 2014; Eissa and Wang, 2016).

Being the most prevalent bacterial pathogen of catfish (Wagner et al., 2006), *E. ictaluri* poses a significant economic threat to the commercial catfish industry (Shoemaker et al., 2009), the most significant cultured finfish in the United States. Antimicrobials applied as a feed additive are the most common means to control ESC. However, anorexia is one of the first clinical signs associated with ESC, limiting the effectiveness of antimicrobial-medicated feed. Also, because *E. ictaluri* can survive in pond mud for an extended period (Plumb and Quinlan, 1986), recurrence of infection is common. Furthermore, antimicrobial treatment may result in emergence of resistant strains (Starliper et al., 1993; Dung et al., 2008).

In Gram-negative bacteria, active transport of nutrients and substrates, including iron, hemin, vitamin B12, carbohydrates, and some transition metal elements are achieved by the TonB complex (TonB-ExbB-ExbD) and TonB-dependent transporters (Schauer et al., 2008; Lim, 2010). The TonB system consists of plasma membrane proteins ExbB-ExbD and periplasmic protein TonB, which provides energy to TonB-dependent receptors to transport substrates across the outer membrane (Liao et al., 2015). The *tonB* gene is located next to *exbB* and *exbD* in the order *exbB, exbD*, and *tonB* in some bacterial species, such as *Neisseria meniningitidis* (Stojiljkovic and Srinivasan, 1997), *Neisseria gonorrhoeae* (Biswas et al., 1997), *Xanthomonas campestris* (Wiggerich et al., 1997), *Pasteurella haemolytica* (Graham and Lo, 1997), and *Helicobacter pylori* (Tomb et al., 1997). In contrast, the *tonB* gene of *Enterobacteriaceae* is not linked to the *exbB* and *exbD* genes (Hannavy et al., 1990; Bruske and Heller, 1993; Bruske et al., 1993).

TonB-mediated active transport of nutrients is critical for survival of pathogenic bacteria during infection (Braun, 2001). Mutation of the *tonB* gene causes attenuation of virulence in several pathogenic bacteria (Jarosik et al., 1994; Seliger et al., 2001; Torres et al., 2001; Bosch et al., 2002; Hsieh et al., 2008). However, there is no information available on the importance of TonB in virulence of *E. ictaluri*. Therefore, the purpose of the current research was to delete the *tonB* gene of *E. ictaluri* and characterize virulence of the resulting mutant (*Ei*Δ*tonB*) in catfish. This study also elucidates the importance of TonB in iron acquisition, which has not been described previously.

## Materials and methods

### Ethics statement

Catfish were used according to a protocol approved by the Institutional Animal Care and Use Committee at Mississippi State University.

### Bacterial strains and growth conditions

*Escherichia coli* C118 λ*pir* (Herrero et al., 1990) was used to clone the in-frame deleted *tonB* gene (Δ*tonB*) into pMEG-375 suicide plasmid (*sacRB mobRP4* R6K *ori* Cm^r^ Amp^r^) (Dozois et al., 2003). *E. coli* SM10 λ*pir* (Simon et al., 1982) was used as the donor strain in conjugation for transfer of the suicide plasmid into wild-type *E. ictaluri* stain 93–146 (Lawrence et al., 1997). Luria-Bertani (LB) and brain heart infusion (BHI) broth and agar (Difco, Sparks, MD) were used to culture *E. coli* at 37°C and *E. ictaluri* at 30°C, respectively. When needed, the following antibiotics and sugars (Sigma-Aldrich, Saint Louis, MN) were added to the culture medium; ampicillin (100 μg/ml), colistin (12.5 μg/ml), sucrose (5%), and mannitol (0.35%).

### In-frame deletion of the *E. ictaluri tonB* gene

The complete open reading frame of the *tonB* gene (locus tag = NT01EI_RS07425) was obtained from the *E. ictaluri* 93–146 genome (GenBank accession: CP001600) (Williams et al., 2012). To delete the *tonB* gene from *E. ictaluri*, gene splicing by overlap extension method was used as previously described (Horton et al., 1989). Briefly, the 1,114-bp upstream and 1,130-bp downstream fragments of the *E. ictaluri tonB* gene were amplified using *EitonB*F01-*EitonB*R42 and *EitonB*F807-*EitonB*R01 primer sets (Table 1), respectively. Fusion of upstream and downstream fragments was accomplished by a second PCR step using *EitonB*F01-*EitonB*R01 primers. The purified Δ*tonB* deletion fragment was cloned into pMEG-375 at the *Sac*I and *Bam*HI restriction sites using T4 DNA ligase (Promega, Madison, WI). Then the resulting plasmid (p*Ei*Δ*tonB*) was transferred into SM10 λ*pir* donor strain and mobilized into *E. ictaluri* by conjugation (Karsi and Lawrence, 2007) to obtain a single crossover strain on BHI agar plates containing ampicillin and colistin. The single crossover strain was streaked on LB agar with 5% sucrose and 0.35% mannitol to allow a second crossover to occur. Mutant verification was performed by ampicillin sensitivity to ensure loss of the plasmid and by PCR using the *EitonB*F01 and *EitonB*R01 primers to confirm Δ*tonB*. Final confirmation was conducted by sequencing the amplified Δ*tonB* fragment using the *EitonB*F01S primer. DNA sequencing was performed by Eurofins (Kentucky, USA).

**Table 1:** List of primers with restriction enzyme used to construct *Ei*Δ*tonB*.

**Primer**	**Sequence 5′-3′**^a^^,^^b^	**RE**
*EitonB*F01	AA**GAGCTC**GTTCAAACGTACCCAACGTGA	*Sac*1
*EitonB*R42	AGCCAGGAAAAATTGCTTCAG	
*EitonB*F807	CTGAAGCAATTTTTCCTGGCTGTGACTGTCTATTTTCGGATCG	
*EitonB*R01	AA**GGATCC**ATGGACTGCCGAATGAAACAA	*BamH*1
*EitonB*F01S	CCTCTGACAGTTCCCAGTTGA	

a *Bold sequences indicate the restriction enzymes (RE) added to the 5′ end primers. Two adenine nucleotides were added to the 5′ to increase the efficiency of restricting cut*.

b *Underlined sequences are the reverse-complement of the EitonBR42 primer*.

*The EitonBF01S primer was used in sequencing of the tonB gene amplified from EiΔtonB*.

### Growth of *EI*Δ*tonB* under iron-depleted conditions

Growth of *E. ictaluri* δ*tonB* and 93–146 were determined in iron-rich medium (BHI broth) and iron-depleted medium as previously described (Holden et al., 2012). Iron depletion in BHI broth was achieved using 100 μM 2′2-dipyridyl (DPD), a ferrous iron chelator (Santander et al., 2012). Growth assays were performed in 24-well plates using a Cytation 5 Cell Imaging Multi-Mode Reader (BioTek, Vermont, USA) at 30°C, with O.D. readings at λ = 600 nm taken every hour for 24 h. All growth experiments were repeated twice. Each experiment was run with six replicates.

### Iron utilization of *Ei*Δ*tonB* under iron-depleted condition

Effects of ferric chloride (FeCl_3_), ferric nitrate Fe(NO_3_)_3_, and ferrous sulfate (FeSO_4_) (Sigma) on the growth of *E. ictaluri* Δ*tonB* and 93–146 were determined under iron-depleted conditions as previously described (Khun et al., 1998). To accomplish this, all iron sources were prepared fresh, sterilized through a 0.45 μ filter, and added to BHI broth at a final concentration of 10 μM. For each iron source, overnight cultures in BHI were adjusted to OD_600_ = 1 before being subcultured at 1:100 into 5 ml BHI media containing 100 μM 2′2-dipyridyl. Absorbance at OD_600_ was measured after 18 h. All growth experiments were performed twice independently with four replicates.

### Assessment of *E. ictaluri* Δ*tonB* virulence

Assessment of virulence was conducted as described (Karsi et al., 2009). Briefly, 240 specific pathogen free (SPF) channel catfish (13.88 ± 0.27 cm and 27.77 ± 1.04 g) were transferred from the SPF fish hatchery at the College of Veterinary Medicine, Mississippi State University to 12 40 L flow-through tanks with aeration (20 fish per tank). Throughout the experiment, fish were kept at 25–28°C and fed to satiety using floating catfish feed. Experimental groups included wild-type strain 93–146, *Ei*Δ*tonB*, and a sham control. Each group was assigned to four tanks randomly. After 1 week acclimation, the water level in tanks was lowered to 10 L. Bacterial cultures grown for 18 h were added to the tanks to provide an infection dose of ~3.32 × 10^7^ CFU per ml of water. CFUs were determined by plating serial dilutions on BHI agar. Fish challenge lasted 1 h, and the sham group was exposed to an equal volume of sterile BHI broth. Fish mortalities were recorded daily. The challenge agent was confirmed as cause of death by culturing anterior kidney swabs on BHI agar. After 21 days post-infection, all fish were re-infected with strain 93–146 (3.83 × 10^7^ CFU/ml water) as described above to evaluate protective immunity. Mortalities were recorded daily, and the mean percent survival for each treatment was calculated.

### Statistical analyses

In iron source utilization experiments, independent variables were time and iron source, while bacterial density (OD_600_) was the dependent variable. Q-Q Plots and the Shapiro-Wilk normality test were used for checking normality of data. Homogeneity of variances was checked using Levene's-Test. One-way ANOVA or Robust-Test of Equality of Means tables were used to determine the presence of significant differences among means (*p* < 0.01). The arcsine transformed percent mortality data were subjected to ANOVA using PROC GLM in SAS for Windows v9.4 (SAS Institute, Inc., Cary, NC) to assess significance. Dunnett's *post-hoc*-test was applied to resolve differences between the means of groups. An alpha level of 0.05 was used in all analyses.

## Results

### In-frame deletion of the *E. ictaluri* Δ*tonB* gene

**Figure 1:**
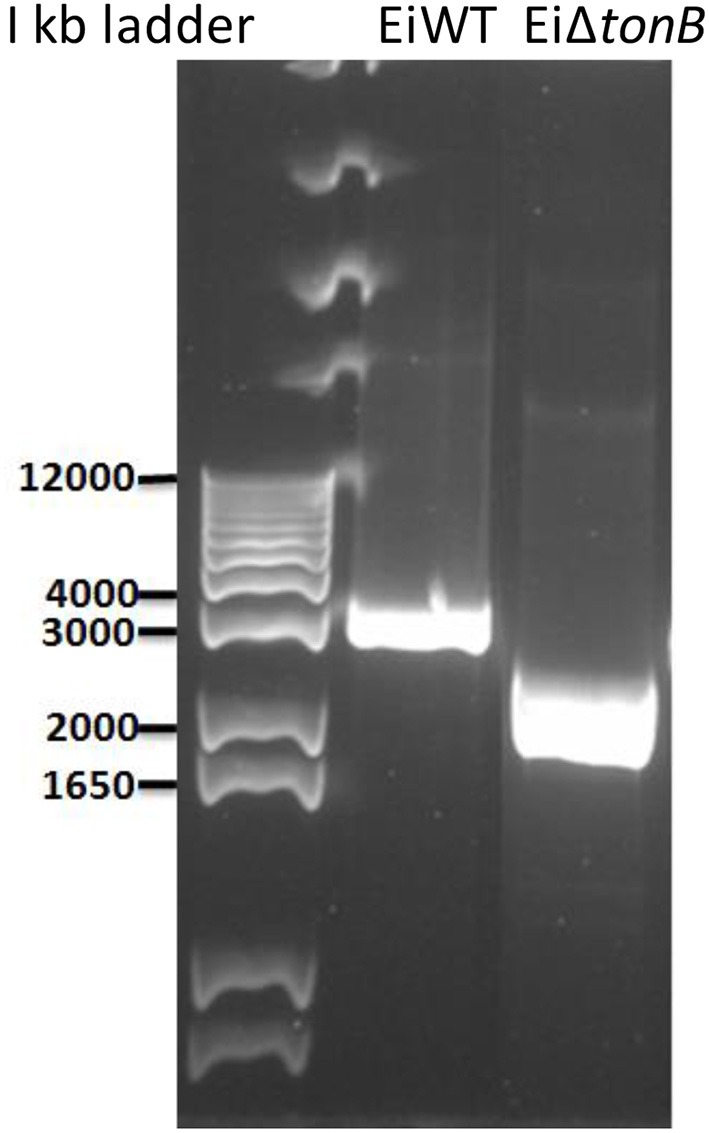
Agarose gel picture showing genotypic confirmation of the *Ei*Δ*tonB* strain by PCR. Lane one is KB Ladder. Lane two is PCR product amplified from wild-type *E. ictaluri*. Lane three is PCR product amplified from genomic DNA of the *Ei*Δ*tonB* strain.

**Figure 2:**
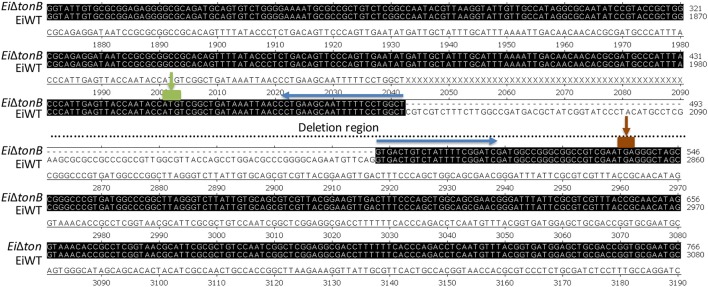
Nucleotide sequence alignment of *tonB* genes in *E. ictaluri* and *Ei*Δ*tonB*. The matching region is shadowed in black. “---” indicates deleted gene region in *Ei*Δ*tonB*. For clarity, the deleted gene region was cropped, which is shown by “…”. The green box and arrow indicate start codon, and the red box and arrow indicate stop codon of the *tonB* gene.

Using a double-selection strategy, we deleted 255 amino acids (including Arg-15 to Gln-269) from the 283 amino acid TonB protein, leaving 14 amino acids at both N- and C-terminals. *Ei*Δ*tonB* construction was confirmed by sequencing the amplified deletion site (Figures 1, 2).

### Growth of *E. ictaluri* Δ*tonB* under iron-depleted conditions

**Figure 3:**
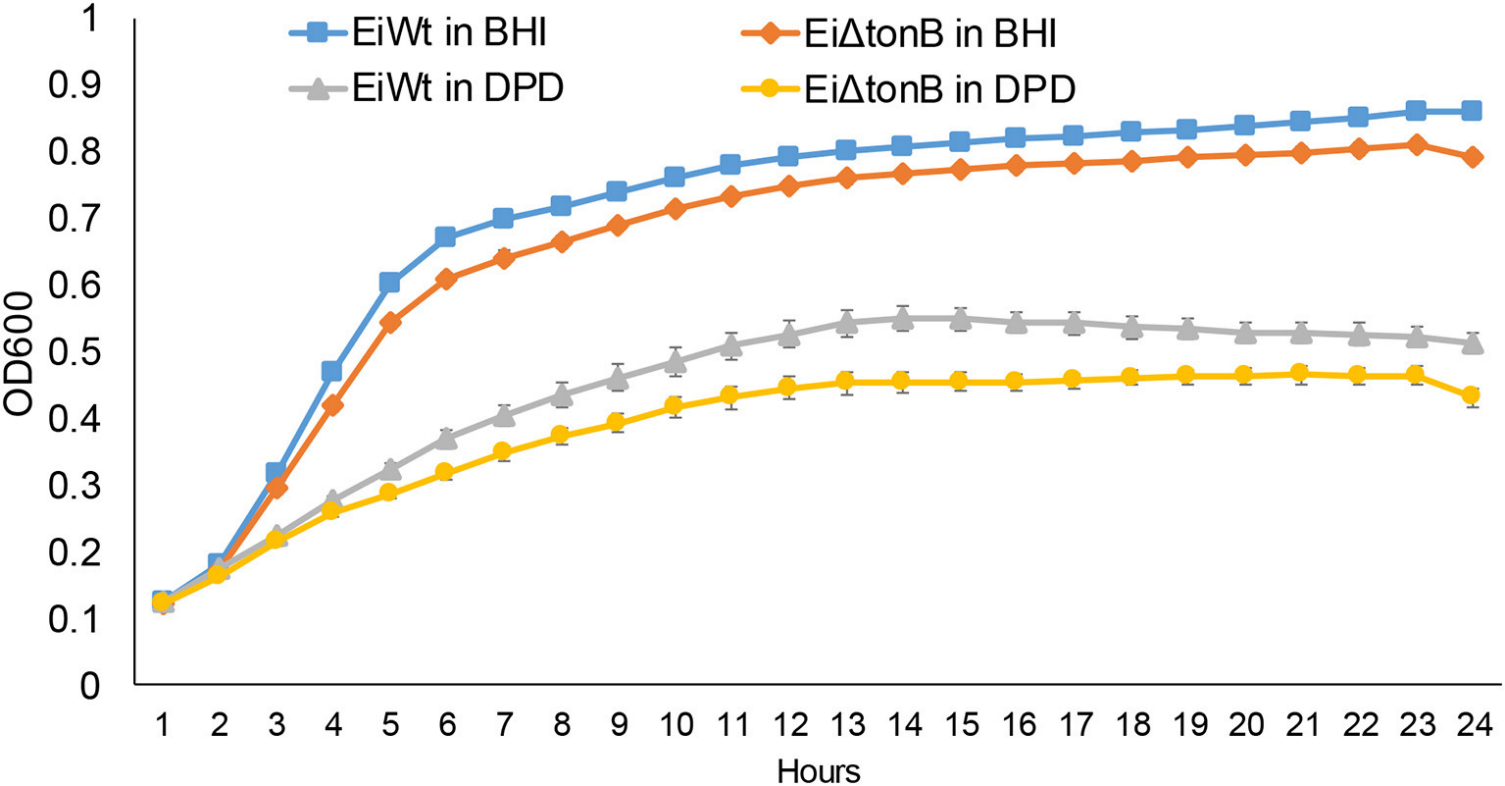
Growth of *Ei*Δ*tonB* and 93–146 in BHI broth and BHI broth supplemented with 100 μM 2′2-dipyridyl (DPD). These data represent the mean ± SE of 12 replicates from two experiments. Standard error bars are shown.

To assess the role of TonB in iron acquisition, we compared the ability of *Ei*Δ*tonB* and wild-type strain 93–146 to grow in standard and iron-depleted BHI broth. Loss of *tonB* caused a significant reduction in growth in standard BHI and when iron was limited by the addition 2,2′-dipyridyl (Figure 3).

### Iron utilization of *E. ictaluri ΔtonB* under iron depleted condition

**Figure 4:**
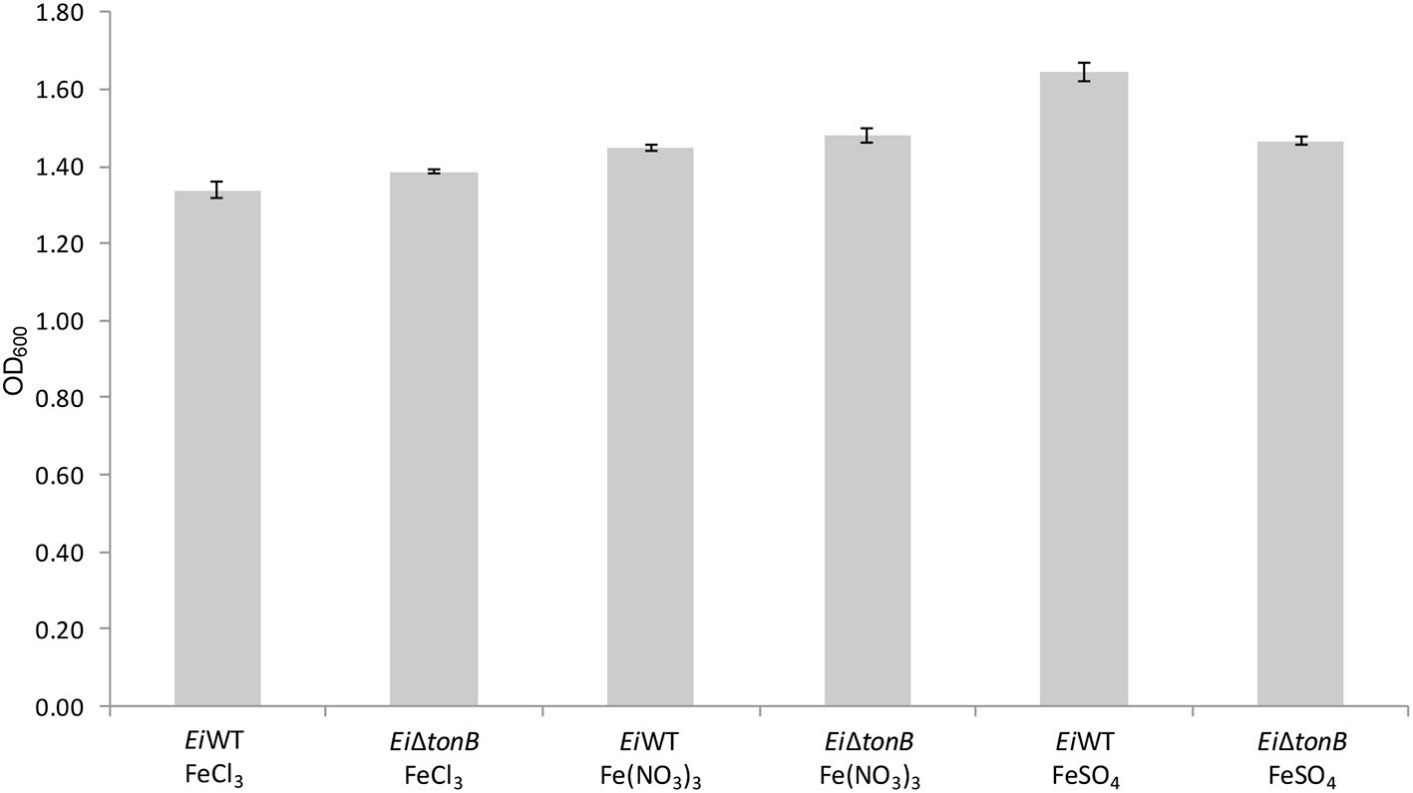
Growth of *Ei*Δ*tonB* and 93–146 in BHI broth containing 100 μM DPD and different ferric iron sources. Data represents the mean ± SE of four replicates.

*Ei*Δ*tonB* was tested for its ability to utilize ferric iron sources in iron-depleted media. There was no significant difference in growth between *Ei*Δ*tonB* and 93–146 in medium containing ferric chloride, ferric nitrate, and ferrous sulfate as a sole iron source (Figure 4).

### Virulence of *E. ictaluri* ΔtonB

**Figure 5:**
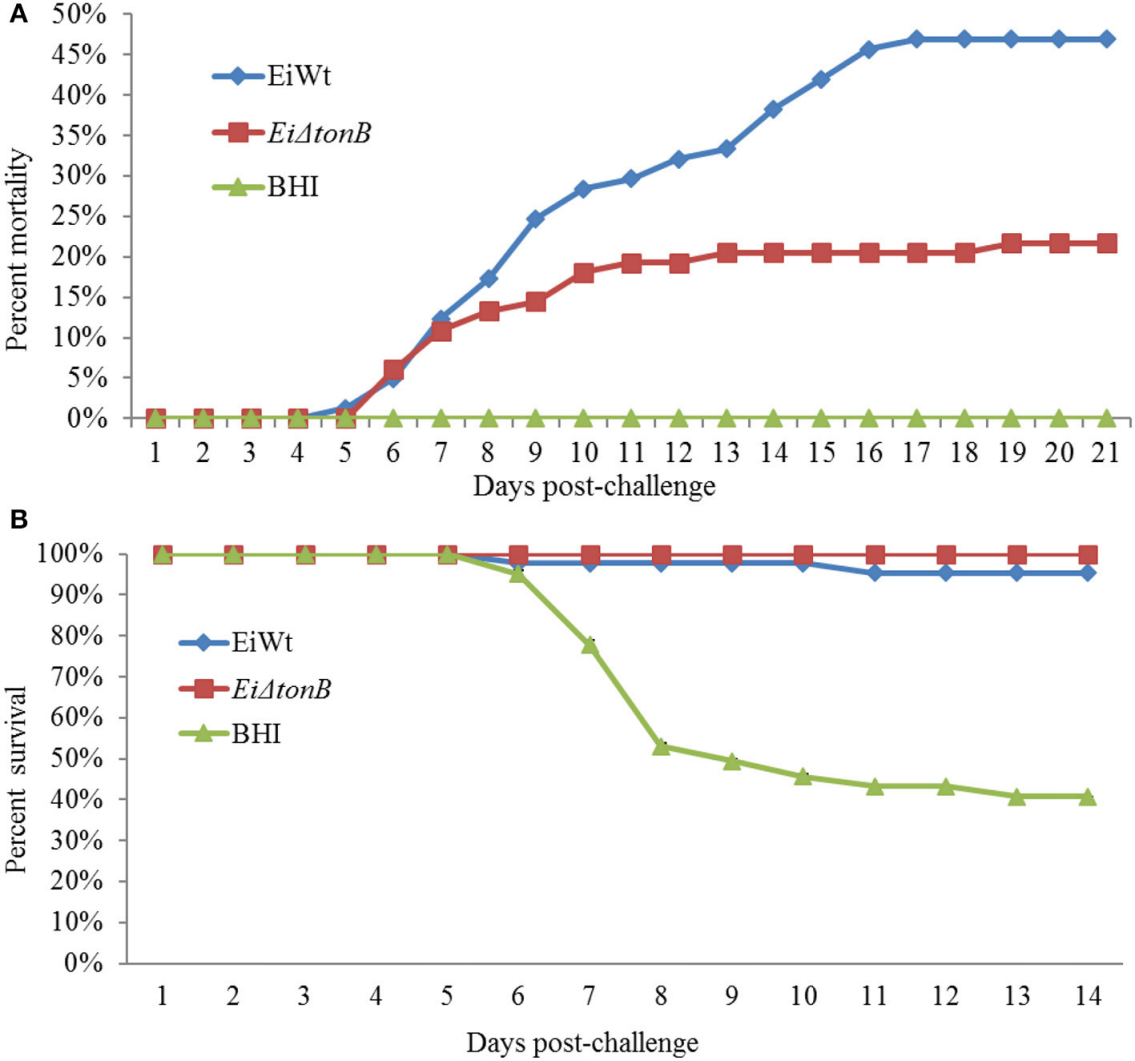
Mean percent mortalities resulting from immersion challenge of *Ei*Δ*tonB* and 93–146 in channel catfish fingerlings **(A)**. Mean percent survival of catfish fingerlings surviving infection with *Ei*Δ*tonB* and re-challenged with 93–146 at 21days post-immunization **(B)**.

Fish infected with *Ei*Δ*tonB* had significantly (*p* ≤ 0.05) lower percent mortalities than fish infected with 93–146 (21.69 vs. 46.91% mortalities) (Figure 5A). At 21 days post-infection, fish surviving *Ei*Δ*tonB* infection had no mortalities when challenged with wild-type strain 93–146, whereas naïve fish had 40.47% mean survival (Figure 5B).

## Discussion

TonB mediates transport of iron and vitamin B_12_, as well as nickel, carbohydrates, and other substrates (Noinaj et al., 2010). In almost all sequenced Gram-negative bacteria, one or more TonB complexes have been identified (Zimbler et al., 2013). The number of TonB proteins is highly variable among bacterial genomes.

The *E. ictaluri* 93–146 genome harbors four open reading frames (ORFs) annotated as TonB-dependent receptors (NT01EI_RS03180, NT01EI_RS07425, NT01EI_RS08370, and NT01EI_RS16830), which are typically involved in transduction of energy for transport of nutrients across the outer membrane. *E. ictaluri* TonB has the highest sequence similarity with *Edwardsiella piscicida* C07-087 TonB (82% identity), *Edwardsiella tarda* FL6-60 TonB (81% identity), and *E. tarda* EIB202 TonB (81% identity), which is in agreement with species phylogeny. The goal of the current research was to elucidate the role of *E. ictaluri* TonB in iron acquisition and virulence.

Iron acquisition and utilization play a central role in bacterial growth. The results of *in vitro* growth assays demonstrated significant decrease in the growth rates in *Ei*Δ*tonB* compared to parent strain 93–146 under both iron-replete and iron-depleted conditions. This suggests that TonB contributes to *E. ictaluri* growth and iron uptake. Interestingly, *E. ictaluri* encodes multiple iron acquisition systems in its genome, indicating the importance of iron uptake and suggesting it is needed during infection. Similar to our findings, mutation of the TonB protein in the fish pathogen *Pseudomonas fluorescens* resulted in decreased growth in LB medium with or without iron supplementation (Hu et al., 2012).

Our results also showed that addition of ferric iron improves growth of both *Ei*Δ*tonB* and wild-type *E. ictaluri*. In a previous study, *E. ictaluri* ferric hydroxamate uptake mutant (*Ei*Δ*fhuC*) was able to grow using various iron sources (Abdelhamed et al., 2013). Multiple TonB systems have been identified in several pathogenic bacteria such as *Vibrio cholera, Vibrio anguillarum, Actinobacillus pleuropneumoniae*, and *P. areuginosa* (Stork et al., 2004). However, not all TonB systems are essential for virulence. For example, in *V. anguillarum*, only *tonB*2 is essential for the transport of ferric anguibactin and virulence; a *tonB*1 mutant is fully virulent (Occhino et al., 1998).

Lack of iron leads to significant stress for bacterial pathogens and is considered a signal that leads to changes in virulence gene expression (Massé and Arguin, 2005). In the gastric environment of catfish, *E*. *ictaluri* encounters iron starvation stress during the initial phase of infection. Our group identified *E. ictaluri* proteins that have increased abundance in iron-restricted conditions (Dumpala et al., 2015). In the present study, catfish experiments demonstrated a 2.16-fold reduction in *Ei*Δ*tonB* virulence compared with wild-type *E. ictaluri*. Similarly, *P. fluorescens* mutants defective in the TonB-dependent outer membrane receptor (TDRs) tdr1, tdr2, and tdr3, which had 26.7, 22.3, and 24.5% mean percent mortalities, respectively, compared with 70% mortality caused by the parent strain in a turbot (*Seophthalmus maximus*) model fish (Zhang et al., 2014).

However, it is possible that the function of TonB in *E. ictaluri* virulence may be distinct from its role in iron acquisition. There is substantial evidence that TonB function is not restricted to iron uptake. *E. ictaluri* TonB could be involved in transport of other substrates or the expression of yet-unidentified virulence factors in the host. The *E. ictaluri* genome does not have *exbB* and *exbD* genes, suggesting that *E. ictaluri* does not utilize the ExbB and ExbD proteins from the TonB-ExbB-ExbD complex. Moreover, deletion of *tolQ* and *tolR* genes, which are *exbB* and *exbD* homologs, does not affect *E. ictaluri* iron utilization (Abdelhamed et al., 2016). In *Shigella dysenteriae*, TonB is required for virulence and growth in the intracellular environment, but it is not required for intracellular iron acquisition (Reeves et al., 2000). Therefore, it is possible that *E. ictaluri* TonB may be required *in vivo* for something other than iron transport.

Catfish surviving infection by immersion with *Ei*Δ*tonB* were completely protected against subsequent infection by the virulent parent strain, indicating that *Ei*Δ*tonB* stimulated a protective immune response. *Ei*Δ*tonB* is not safe to be considered a live attenuated vaccine candidate, but our results demonstrate that deletion of TonB causes attenuation without affecting protective immunogenicity. Therefore, it could be a viable gene to use in combination with other gene deletion(s) to develop a live attenuated vaccine.

In conclusion, our experiments showed that TonB participates in virulence of *E. ictaluri* and contributes to optimal host infection. To our knowledge, this study is the first to describe the contribution of TonB to *E. ictaluri* virulence. Further work is required to determine which iron transport system or combinations of systems are used to acquire iron during *E. ictaluri* infection.

## Author contributions

HA, ML, and AK planned the experiments. HA and AK performed the experiments and analyzed the data. HA, ML, and AK wrote the manuscript.

### Conflict of interest statement

The authors declare that the research was conducted in the absence of any commercial or financial relationships that could be construed as a potential conflict of interest.
